# Lipid and Peptide-Oligonucleotide Conjugates for Therapeutic Purposes: From Simple Hybrids to Complex Multifunctional Assemblies

**DOI:** 10.3390/pharmaceutics15020320

**Published:** 2023-01-18

**Authors:** Carme Fàbrega, Anna Aviñó, Natalia Navarro, Andreia F. Jorge, Santiago Grijalvo, Ramon Eritja

**Affiliations:** 1 Nucleic Acids Chemistry Group, Institute for Advanced Chemistry of Catalonia (IQAC-CSIC), Jordi Girona 18-26, E-08034 Barcelona, Spain; 2 Networking Center on Bioengineering, Biomaterials and Nanomedicine (CIBER-BBN), Jordi Girona 18-26, E-08034 Barcelona, Spain; 3 Department of Chemistry, Coimbra Chemistry Centre (CQC), University of Coimbra, Rua Larga, 3004-535 Coimbra, Portugal; 4 Colloidal and Interfacial Chemistry Group, Institute for Advanced Chemistry of Catalonia (IQAC-CSIC), E-08034 Barcelona, Spain

**Keywords:** antisense oligonucleotides, siRNA, lipid-oligonucleotide conjugates, peptide-oligonucleotide conjugates

## Abstract

Antisense and small interfering RNA (siRNA) oligonucleotides have been recognized as powerful therapeutic compounds for targeting mRNAs and inducing their degradation. However, a major obstacle is that unmodified oligonucleotides are not readily taken up into tissues and are susceptible to degradation by nucleases. For these reasons, the design and preparation of modified DNA/RNA derivatives with better stability and an ability to be produced at large scale with enhanced uptake properties is of vital importance to improve current limitations. In the present study, we review the conjugation of oligonucleotides with lipids and peptides in order to produce oligonucleotide conjugates for therapeutics aiming to develop novel compounds with favorable pharmacokinetics.

## 1. Introduction

Over the last decade, therapeutic oligonucleotides have gained momentum as an approach to drug development; consequently, there has been a large development of the field. Although the first oligonucleotide approved for therapeutic application in humans dates back to 1998 [1], the recognition of their full therapeutic potential started in 2016 with the authorization of Spinraza [2], for the treatment of Spinal muscular dystrophy, and Etiplersen [3], for the treatment of Duchenne muscular dystrophy. In both cases, the possibility of targeting a mutated gene through alternative splicing became a major success for a long-time dream. Since then, the list of oligonucleotides approved for human practices has reached the dozens, especially with the incorporation of siRNAs in the therapeutic arena [4].

Oligonucleotide therapeutics include antisense oligonucleotides (ASOs) [5], small interfering RNAs (siRNAs) [4], aptamers [6], microRNAs [7], and others [8]. ASOs are small single stranded nucleic acids that by complementarity, bind to a particular mRNA and form a hybrid molecule to modulate gene expression. They act through two mechanisms of action (a) by steric blockade at the ribosomes or (b) by recruiting RNase H enzyme that catalyzes the degradation of mRNA [5]. On the other hand, siRNAs consist of 21–23 mer RNA duplex formed by a sense and an antisense strand complementary to mRNA. The latter is responsible for the recruitment of the target transcript into the RNA-induced silencing complex (RISC) that leads to gene silencing [4]. Unmodified oligonucleotides are not readily taken up into tissues and are also susceptible to degradation by nucleases. For these reasons, the design and preparation of more stable modified DNA/RNA derivatives to improve the existing limitations, like inefficient delivery and mature to the position of clinical utility, is of key importance. Regarding the nuclease resistance, novel derivatives are being developed [9]. The delivery issue is being addressed by the following approaches: by encapsulation in nanomaterials such as solid lipid nanoparticles (SLNs) or by preparing novel oligonucleotide conjugates with selective targeting moieties [10]. The first FDA-approved siRNA, Onpattro [11], is the paradigm of the former; *N*-acetylgalactosamine (GalNAc) oligonucleotide conjugates [12] are the paradigm of the latter. GalNAc oligonucleotide-conjugates have been shown to be delivered to hepatocytes by binding to asyaloglycoprotein receptors [12]. The latest FDA-approved therapeutic siRNAs, including Givosiran [13], Lumasiran [14], Inclisiran [15], Vutrisiran [16], are based on this strategy. Importantly, Inclisiran (Leqvio^®^) exerts its therapeutic action within a twice-yearly administration regime [17] while most of the therapeutic siRNAs are administered monthly or every two months [13,14,16]. The large duration of the therapeutic effects of Inclisiran is not only due to a combination of the stability achieved by the modifications on the siRNAs and the efficacy in the delivery, but also to the efficient inhibition of the proprotein convertase subtilisin kexin type 9 (PCSK9) [15,17].

The success in exploring oligonucleotide conjugates for hepatic delivery has triggered an intense quest for oligonucleotide conjugates with tissue-selective targeting properties, particularly for extrahepatic delivery [18]. In this review, we provide an overview of major developments on the preparation of lipid and peptide conjugates. At the beginning of the antisense strategy, these conjugates had already been explored. They have regained attention recently as extensive effort is being made to evaluate them on RNA interference mechanisms and, in general, on new discoveries in the RNA field [19] to meet unsolved and emerging clinical needs.

## 2. Results and Discussion

### 2.1. Early Developments in the Synthesis of Lipid-Oligonucleotide Conjugates

**Figure 1:**
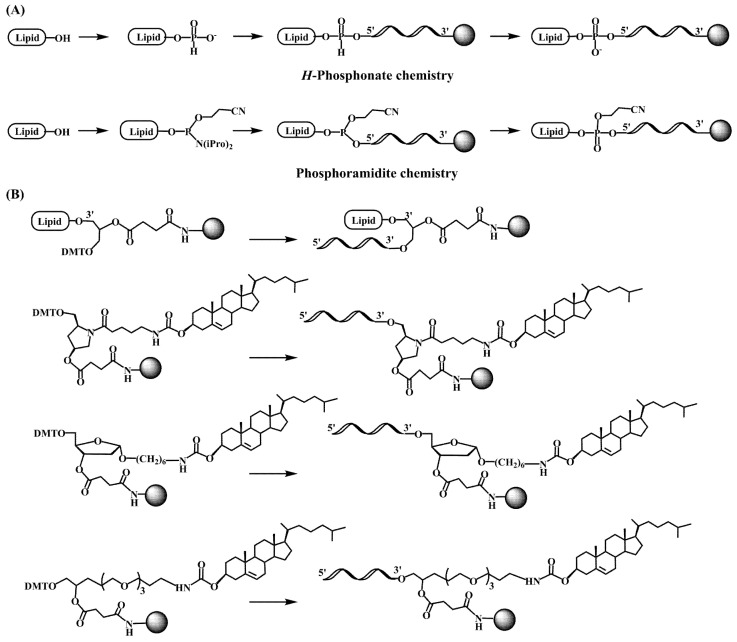
Synthetic approaches for the incorporation of lipids to the 5′ and 3′ end of an oligonucleotide. (**A**) *H*-phosphonate [22,23,24] and phosphoramidite chemistry [29,31] and (**B**) Different cholesterol functionalized solid supports [30,32].

The pioneering works on the antiviral activity of oligonucleotides [20,21] stimulated the development on lipid-oligonucleotide conjugates as potential candidates for the inhibition of the human immunodeficiency virus (HIV-1) in cell culture. Cholesterol was first selected to enhance the interaction between oligonucleotides and cell membranes, which increases the antiviral activity of the oligomers [22,23]. Letsinger’s group designed a synthetic protocol based on the solid-phase oxidation of *H*-phosphonate dinucleotide intermediates with amino-functionalized cholesterol and catalyzation by carbon tetrachloride, which generated the desired cholesterol-oligonucleotides bond through a phosphoramidate link (Figure 1A) [22] or by direct coupling at the 5′-termini with the *H*-phosphonate derivative of cholesterol [23,24]. The *H*-phosphonate derivative of a diacylglycerol was also used for the incorporation of 1,2-di-*O*-hexadecyl-*rac*-glyceryl residue at the 5′-end of antiviral oligonucleotides [25]. Solution techniques using amino-lipids [26] or thiocholesterol [27] were also used for conjugation in order to generate physiologically-labile ester [26] or disulfide [27,28] bonds between the lipid and the oligonucleotide. These groundbreaking studies proved the utility of lipid-oligonucleotides by demonstrating that the lipid moiety enhances nuclease resistance and maintains or improves hybridization properties [25,29]. However, in some cases, antiviral properties groundbreaking antisense inhibition rules suggest other mechanisms, such as binding to viral and/or cell membranes [22,23,25].

**Scheme 1:**
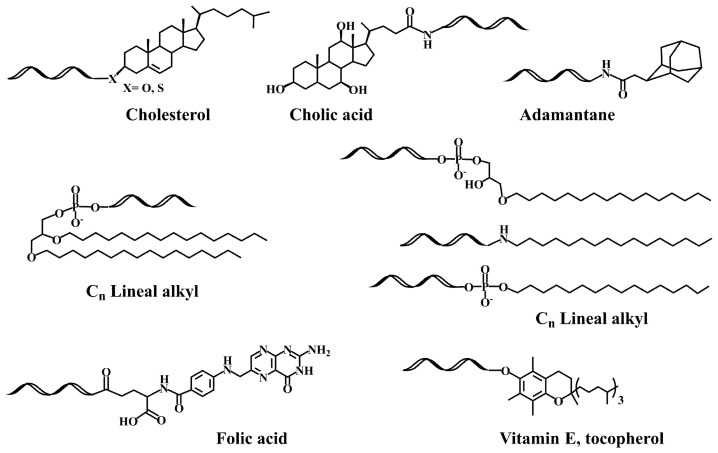
Structure of several lipid-oligonucleotide conjugates [31,32,35,36,37,39,40].

The next step was the development of specific lipid-phosphoramidites and lipid-functionalized solid supports (Figure 1B). Due to the lability of the ester bonds to ammonia [30], they were replaced by ether, amide and urethane linkers. Several derivatives carrying ether and glyceryl ether bonds were developed by the group of Tom Brown, including 3′ and 5′-cholesteryl, 5′-(1,2-dihexadecylglyceryl), 3′ and 5′-hexadecyl, 5′-octadecyl and 5′-adamantyl [31] as well as vitamin E derivatives (Scheme 1) [32]. Other groups worked on new cholesterol derivatives containing aminodiols such as 3-amino-1,2-propanediol [29] and 3-aminopropylsolketal [33,34], in which the cholesterol moiety was linked to the amino group by reaction with cholesterol chloroformate generating an urethane bond stable to ammonia.

On the other hand, postsynthetic conjugation reactions between amino-oligonucleotides and carboxylic acid derivatives of lipids such as cholic acid, adamantane acetic acid and fatty acids were described [35,36,37]. A variation of this protocol implies the addition of 9-fluorenylmethoxycarbonyl (Fmoc)-protected amino linkers into oligonucleotides. After the assembly of the sequence, the Fmoc moiety can be removed generating a free amino group that reacts with cholesterol chloroformate followed by standard ammonia deprotection [38].

The availability of lipid-oligonucleotides allowed its preclinical evaluation demonstrating specific antisense activity, enhanced nuclease resistance and maintenance or improvement of hybridization properties [41,42]. Another interesting property is its capability to bind to serum proteins and lipoproteins, which is important to avoid renal clearance of oligonucleotides [43]. Several specific receptor-mediated uptake mechanisms have been described to explain selective uptake by hepatocytes via lipoprotein receptors [44]. Although lipid-oligonucleotide conjugates have interesting properties, none of them, except polyethyleneglycol (PEG) derivatives, have found their way to clinical studies. The synthesis of oligonucleotides functionalized with PEG has been described by the Erdmann and Bonora groups, being somehow similar to the methodology described in this section for other lipid diols [45,46,47,48]. Pegaptanib (Macugen^®^) is a therapeutic oligonucleotide used for the treatment of aged-associated macular degeneration. This oligonucleotide is an aptamer constituted by 28 nucleotides and functionalized with PEG at the 5′-end and an inverted-T at the 3′-end to prevent degradation by nucleases [49]. This aptamer has a strong affinity to the vascular endothelial growth factor VEGF165 (Kd = 49 pM), inhibiting the binding of VEGF to its receptor, suppressing the VEGF-mediated angiogenesis and consequently lowering vascular permeability and inflammation [50].

### 2.2. Lipid-Oligonucleotide Conjugates and the Development of RNA-Based Therapeutics

The discovery of the RNA interference mechanisms provided a great resurgence in the area of therapeutic oligonucleotides. Soon after the work of Mello and Fire [51], it was established that the effector molecules of the RNA interference process were double stranded RNA molecules of 19–21 nucleotides. Then, synthetic oligonucleotides with chemical modifications were proved to improve the efficacy and the duration compared to natural substrates [52,53]. Later, the discovery of the microRNAs increased the therapeutic potential of oligonucleotides [54]. Cholesterol-siRNAs were developed and were demonstrated to be successful derivatives for the inhibition of lipoproteins [55]. Stable nucleic acid lipid nanoparticles (SNALP) and solid-lipid nanoparticles (SLN) were developed for the delivery of siRNAs [56,57] showing for the first time the in vivo inhibition of *ApoB* in non-human primates [56]. To expand the arsenal of available cationic lipids for siRNAs delivery, several lipids and lipoids libraries were screened, thereby generating lipids with high efficiency and less toxicity [58,59].

These studies led to the search for new hydrophobic molecules to enhance the cellular uptake of siRNAs [60,61,62,63]. Conjugation of amino-siRNAs with a small library of carboxyl-lipids including cholesterol, fatty-acids and bile acids resulted in hydrophobic siRNA derivatives that interact with lipoproteins [44]. The obtained hydrophobic (lipo-siRNA-protein) complexes were efficiently delivered to liver, gut and kidney by specific lipoprotein-mediated receptors. Inspired by these results, we studied a small lipid library including both neutral [39] and cationic lipids [40]. The study of TNF-alpha inhibition with and without lipofectamine proved that these lipid-siRNA conjugates carrying ammonia-resistant glycerol ether bonds were compatible with RNA interference mechanisms. A lipid carrying two linear hydrocarbon chains was the best derivative in terms of increasing cellular entrance (Scheme 1) [39]. These double-chain lipid-siRNA conjugates stimulated the formation of small vesicles that may explain the improved uptake properties [64,65]. In addition, a good correlation was found among cell lines expressing abundant CR3 receptors [64]. The vesicle formation properties and the enhanced binding of siRNAs carrying double-chain lipids to hydrophobic membranes has also been observed by several authors [66,67,68]. Furthermore, we found that the sonication of lipophilic siRNA in presence of serum enhanced the binding of lipophilic-siRNA to lipoproteins, resulting in a more efficient transfection [69].

**Figure 2:**
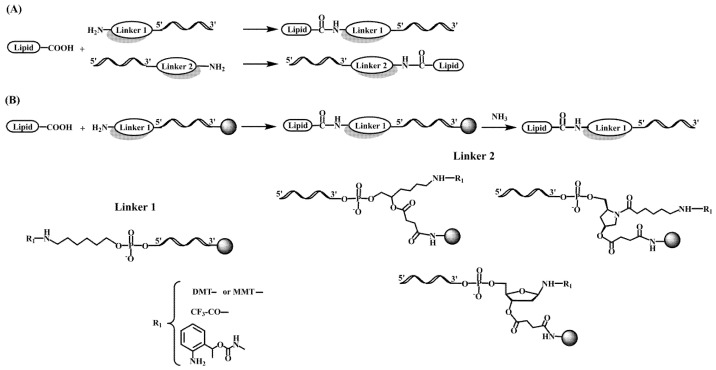
Chemical structure of several linker molecules connecting solid supports and lipids used for the preparation of lipid-oligonucleotide conjugates. (**A**) Lipid conjugation to the 5′ or 3′ end of an oligonucleotide in solution [35,36,37]. (**B**) Lipid conjugation to the 5′ or 3′ terminus of an oligonucleotide on a solid support [29,31].

In a different approach, cholesterol-conjugated single-stranded short RNA molecules, or antagomiRs, were successfully used to silence miRNA [70,71,72,73]. In addition, G-quadruplex-forming oligonucleotides carrying lipid moieties were found to increase their affinity for viral membrane proteins showing antiviral properties by inhibition of viral cell entry [74,75,76]. The most frequent methodology for the preparation of oligonucleotide-lipid conjugates is based on amide formation (Figure 2), but other reactions such as the copper catalyzed azide-alkyne cycloaddition (click chemistry) have also been reported [77].

**Figure 3:**
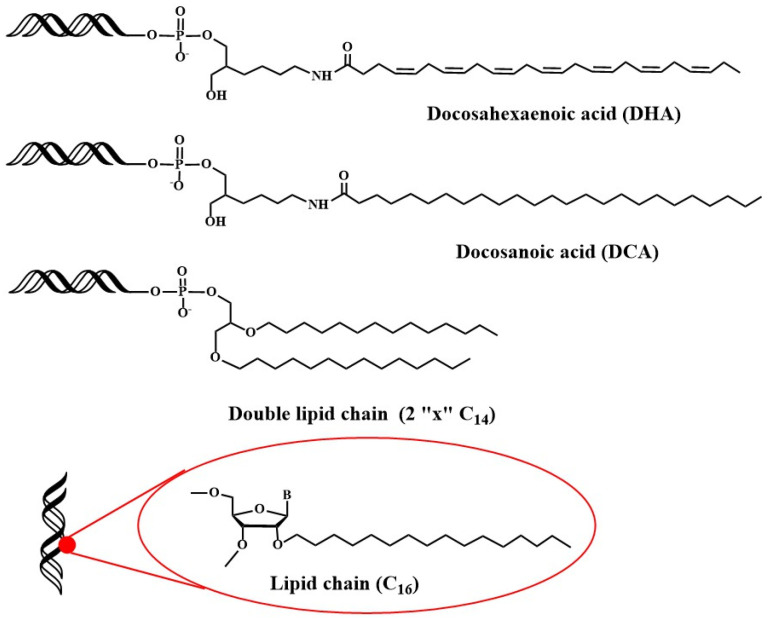
Structure of four different lipid-siRNAs that have been successfully delivered to different cell types and organs [81,92].

Recently, studies have been addressed towards the application of lipid-oligonucleotides to transfect cells and tissues other than liver. Primary neurons are difficult to transfect with siRNAs because the unique nature of the blood brain barrier (BBB) and the difficulty of direct administration [78]. Alterman et al. found that cholesterol-tetraethyleneglycol functionalized siRNA at the passenger strand was efficiently internalized in primary cortical neurons, inducing a potent and specific silencing of huntingtin gene [79,80]. This potent silencing activity was maintained in vivo when injected into mouse brain [79]. Additionally, docosahexaenoic acid conjugation (Figure 3) was judged to increase further the distribution and the inhibitory properties of lipid-siRNAs when administered into the brain [81].

Another interesting property of lipid-siRNAs is the enhancement of siRNA loading into extracellular vesicles [82,83], which generates attractive nanoparticles for the delivery of therapeutic siRNAs. The best option in terms of higher loading and efficiency was the conjugation of vitamin E (Sheme 1) [83]. Recently, siRNAs were modified with vitamin E by a benzonorbonadiene linker, which releases active siRNAs when reacting with tetrazines [84].

Next, the distribution of siRNAs conjugated to a small library of complex lipids was analyzed, including saturated and unsaturated fatty acids, steroids and lipophilic vitamins with or without phosphocholine heads. The level of hydrophobicity is critical in order to define accumulation in the liver or in the kidney. In addition, it was shown that some lipid derivatives were able to accumulate in non-hepatic tissues such as lung, muscle, heart, adrenal glands and fat [85,86]. In more detailed studies, factors such as the chemical structure of the lipids [86], the phosphorothioate content [87], the presence of single-stranded phosphorothioate regions [88] or the valency of fatty acid modifications [89] were demonstrated to affect the pharmacokinetics, the extrahepatic distribution and the in vivo efficacy of lipid-siRNAs [90,91]. Recently, the in vivo properties of siRNA carrying 2′-*O*-hexadecyl (C16) moieties have been described (Figure 3). These lipophilic siRNAs can be delivered into the central nervous system, eye and lungs of rats and non-human primates, where they exert inhibitory properties for at least 3 months [92]. These results opened the possibility of using lipophilic siRNAs in the treatment of Alzheimer’s disease.

A systematic study on the effect of the conjugation of antisense oligonucleotides with fatty acids confirms its potential delivery to muscle and other extrahepatic tissues [93,94]. Moreover, palmitic acid-, tocopherol-, and cholesterol-conjugated (Scheme 1) antisense oligonucleotides were reported to increase protein binding and enhance intracellular uptake [95]. These properties were explored by several groups for the development of lipid-antisense oligonucleotides targeting the exon 51 of human Duchene Muscular Distrophy gene [96,97].

The development of mRNA vaccines, especially during the COVID-19 pandemic, triggered the interest in lipid nanoparticles for mRNA delivery. The approval of Onpattro for the treatment of transthyretin–mediated amyloidosis demonstrated the efficacy and safety of these non-viral vectors for siRNA delivery to liver [11]. This approval facilitated the rapid authorization of the two mRNA vaccines for SARS-CoV-2 [98,99]. The great potential of mRNA vaccines for cancer, infectious diseases and genetic disorders is stimulating the search for the next generation of lipid nanoparticles that would increase efficacy, degradability [100] and tissue-specificity properties [101].

### 2.3. Early Developments in the Synthesis of Oligonucleotide-Peptide Conjugates

**Figure 4:**
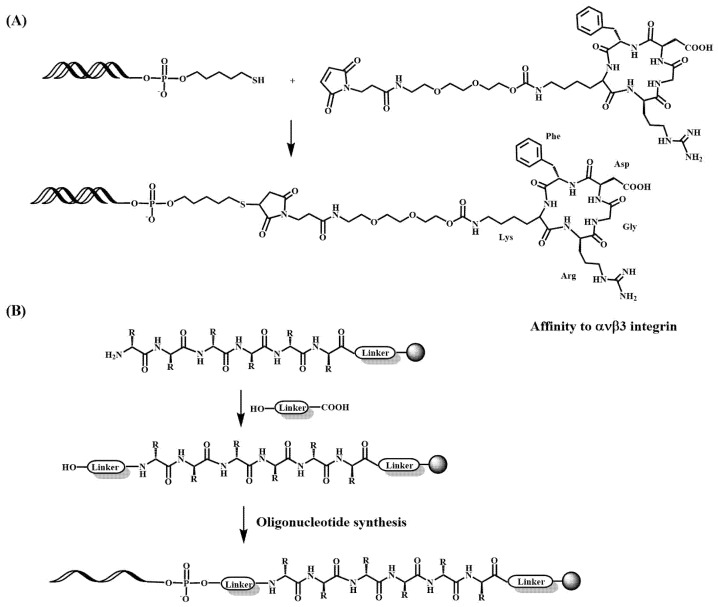
Preparation of oligonucleotide-peptide conjugates. (**A**) Postsynthetic conjugation of a thiol-oligonucleotide with a peptide by a maleimide moiety [124]. (**B**) Stepwise method for the synthesis of oligonucleotide-peptide conjugates on a solid support [113,114,115,116].

Peptides can be used to improve the potency of therapeutic oligonucleotides by conferring tissue and attaching cell-targeting, cell-penetrating or antiviral and antibacterial properties to them. The cellular internalization mechanisms of peptides may be divided in two main pathways: direct penetration or translocation, i.e. energy independent or energy-dependent endocytosis [102]. The energy-independent mechanisms are described for peptide/oligonucleotides non-covalent complexes at high peptide concentration. While most of the oligonucleotide-peptide conjugates use the natural energy-dependent process, this one involves encapsulation of the cargo in membrane vesicles. Depending on the nature and size of the conjugate, it can be classified as macropinocytosis, clathrin- or caveloin-mediated endocytosis as well as clathrin/caveloin- independent endocytosis [103]. Once internalized, an important issue is to facilitate the endosomal escape to avoid degradation of the conjugates. Some peptides introduce pH-sensitive domains for the destabilization of the membranes and allow the release of the conjugates into the cytosol [103]. One of the first examples of this was demonstrating that the conjugation of oligonucleotides complementary to the vesicular stomatitis virus (VSV) to poly(L-lysine) had increased antiviral properties than unmodified oligonucleotide [104,105]. Then, defined peptides carrying the Lys-Asp-Glu-Leu (KDEL) peptide [106] and the Lys-rich SV-40 large T-antigen nuclear localization sequences [107] were incorporated into oligonucleotides. In these cases, the thiol-oligonucleotides reacted with peptides carrying maleimide or Cys [106,107,108] residues in a postsynthetic conjugation (Figure 4A). Several variations have been described [109], including the reaction of thiol-oligonucleotides with bromoacetyl-peptides [110] or iodoacetamide- or maleimide oligonucleotides with thiol-containing peptides [111,112].

**Figure 5:**
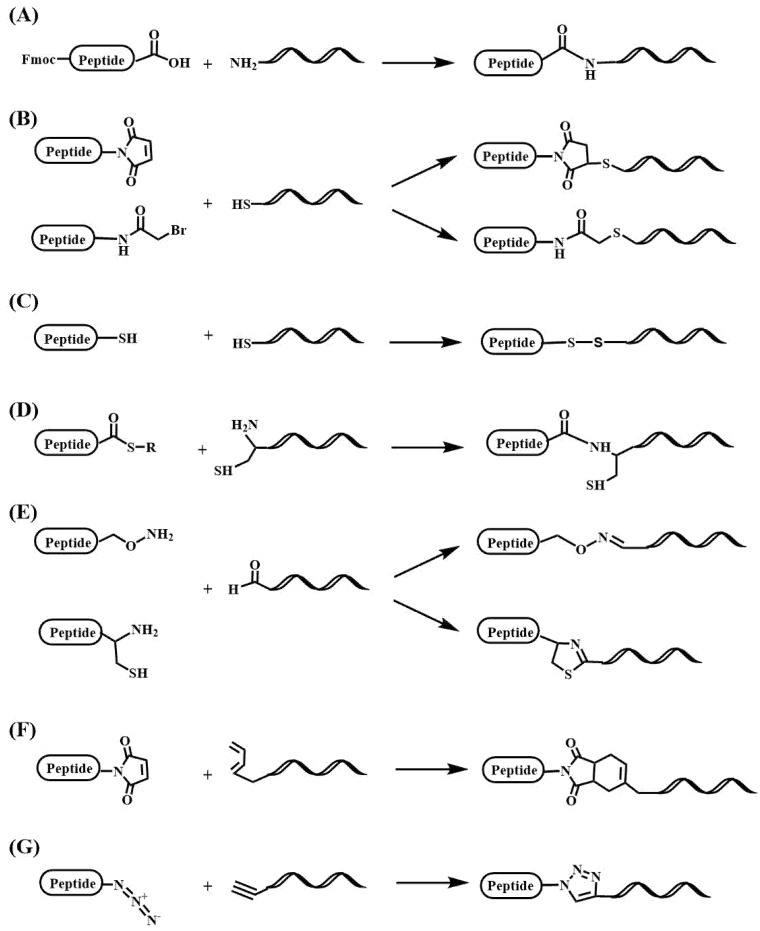
Postsynthetic reactions for the synthesis of oligonucleotide-peptide conjugates. (**A**) Using protected peptide segments for fragment condensation [175], (**B**) thiol-maleimido and thiol-bromoacetamido reactions [176], (**C**) disulfide formation [177,178,179], (**D**) synthesis by native ligation [180], (**E**) oximes or thiozolidine reactions [181,182,183], (**F**) conjugation by Diels-Alder reaction [184,185], (**G**) conjugation reactions catalyzed by copper to produce alkyne-azide cyclo additions [186,187,188].

Thereafter, stepwise methods for the synthesis of the conjugates using one single solid support were developed [113,114,115,116] (Figure 4B). Usually, the peptide moiety is first assembled using *t*-butoxycarbonyl (Boc)-amino acids with base labile protecting groups, avoiding the use of strong acids in the presence of the oligonucleotide [115,117]. However, in some cases Fmoc-amino acids protected with the Boc- [117,118] or the 1-(4,4-dimethyl-2,6-dioxocyclohex-1-ylidene) ethyl (Dde) [114] groups have been described. The study of appropriate Fmoc-protected amino acids for trifunctional amino acids has been carefully analyzed by several groups [119,120,121,122]. The preparation and condensation of protected peptide fragments has also been used in the preparation of peptide-oligonucleotide conjugates (Figure 5A) [123]. This approach allows the incorporation of the peptide at the 5′-end in one single coupling reaction, thus avoiding repetitive deblocking steps.

Oligonucleotides linked to cell penetration peptides (CPP) are among the most studied conjugates [103,125] (Table 1). These short peptides can pass through cell membranes, facilitating the intracellular transport of various payloads. They can be: polycationic, with examples including Arg-rich [126,127], Tat peptide [128], Penetratin [129,130]; amphipatic, for example Transportan [131], MAP-peptide [132]; proline-rich [133,134]; or hydrophobic, like C105Y [135], Pept1 [136] or MPM-peptides [137]. A similar strategy was developed consisting in the derivatization of antisense oligonucleotides with peptides that are recognized by membrane receptors, such as RGD peptides with affinity to integrins [138] or octreotate derivatives with affinity to somatostatin receptors [139]. Interestingly, these strategies combined with the addition of fusogenic peptides help endosomal escape [138].

### 2.4. Peptide-Oligonucleotide Conjugates and the Development of RNA-Based Therapeutics

Peptides are an attractive source of ligands being that its conjugation to oligonucleotide-siRNAs is of special interest. Table 1 shows some of the most advanced peptide sequences described for the delivery of therapeutic oligonucleotides. A large number of these peptides are amphipathic peptides with the ability of self-assembling into NPs, to which oligonucleotides are associated by electrostatic or hydrophobic interactions [165,166,167,168,169,170,171,172,173].

In addition, siRNAs have been directly conjugated to peptides. The lability of siRNAs to basic conditions and the protection of the 2′-OH created extra challenges the preparation of RNA-peptide conjugates. Although there is some work describing the preparation of siRNA-peptide conjugates by stepwise synthesis [175], most of the protocols are based on postsynthetic conjugation (Figure 5). The first ones described the use of thiol-maleimide reactions (Figure 4A and Figure 5B) [176] or disulfide formation (Figure 5C) [177,178,179]. The following ones define a large variety of novel postsynthetic reactions, including native ligation (Figure 5D) [180], formation of oxime (Figure 5E), thiazolidine or hydrazone bonds [181,182,183], Diels-Alder (Figure 5F) [184,185] and alkyne-azido click reactions (Figure 5G) [186,187,188]. All these studies generated a large number of specialized phosphoramidites and functionalized solid supports to produce the desired oligonucleotides carrying reactive groups, such as: amino, thiol, carboxylic, alkyne, alkene, aldehyde and azido (Figure 5). These methodological advances can be found in recent reviews [103,189,190,191].

Another type of peptide known as homing peptides were developed by phage display technology. These peptides were successfully used to deliver antisense oligonucleotides to cardiac tissue [140] (Table 1), or siRNAs [192] and DNA plasmids [193] to spinal cord or to microglia. Centyrins are small proteins that can be redesigned to bind numerous antigens increasing extrahepatic delivery. Centyrins-siRNA conjugates have been shown to improve tumor delivery and tumor regression [194].

**Figure 6:**
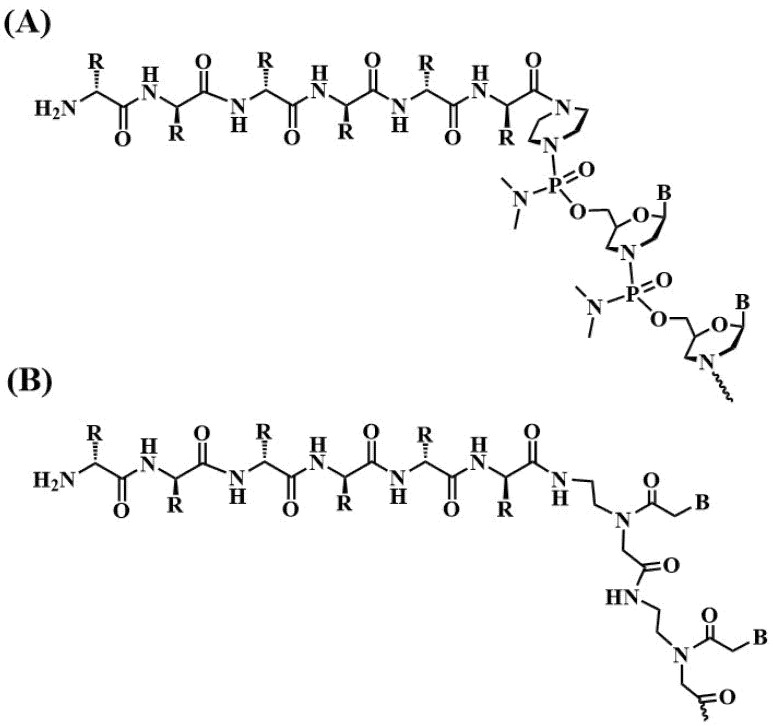
Chemical structure of nucleic acid conjugates for potential therapeutic use. (**A**) Phosphorodiamidate morpholino oligomers (PMO) [197] and (**B**) peptide nucleic acids (PNA) conjugates [198].

Antisense oligonucleotides can also selectively bind to immature mRNAs to redirect splicing. The design of antisense oligonucleotides complementary to splice regions has received much attention due to its ability to create steric blocks to permit the binding of splicing factors of the immature mRNA. Exon skipping is based on the observation that excluding out-of-frame exons generates truncated but partially functional proteins instead of harmful proteins. This is the mechanism of Eteplirsen [3] and other antisense oligonucleotides approved for neurological disorders. In this particular strategy, the modification of ASOs with phosphorodiamidate morpholino oligomers (PMO) is frequently exploited. The conjugation of peptides to PMOs (Figure 6A) is being intensively studied for the treatment of various muscular dystrophies, most notably for Duchenne muscular dystrophy (DMD) (Table 1). For this reason, several peptide libraries have been screened and various peptide-PMOs are being validated in preclinical studies [165,195,196].

Recently, several peptides such as EDO (enhanced delivery oligonucleotide, PepGen), and SRP-5051 (Sarepta) [197] are being analyzed for the treatment of DMD.

Other types of nucleic acid conjugates being investigated are peptide nucleic acids (PNA, Figure 6B) linked to peptides, especially CPP conjugates, as promising antibacterial agents [198]. The assembly of peptides on PNA oligomers is done by stepwise synthesis on the same support as both PNA and amino acid monomers which have similar protecting group schemes [198,199]. This topic has special interest as the number of bacteria resistant to antibiotics is growing dangerously; therefore, nucleic acid derivatives have an important role for the gene-specific bacterial control.

### 2.5. Multifunctional Conjugates

**Figure 7:**
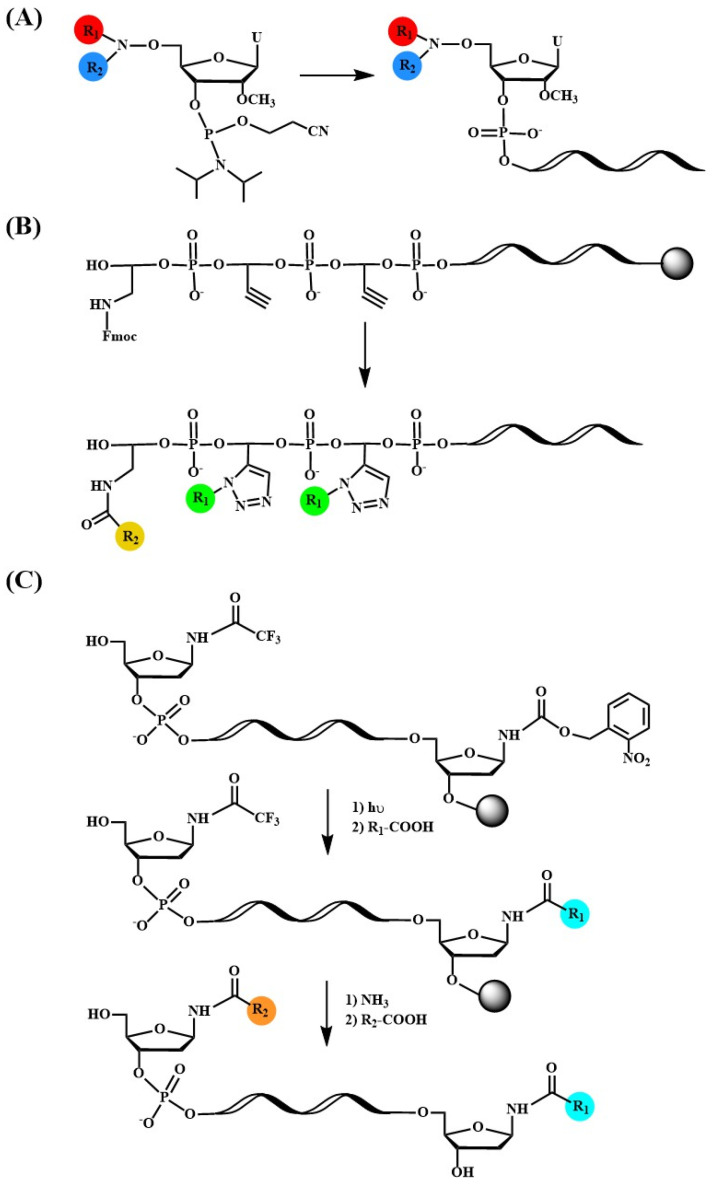
Chemical synthesis of multifunctional peptide and/or lipid oligonucleotide conjugates. (**A**) Modified phosphoramidite for the incorporation of two ligands, (**B**) combination of amino-protection and click chemistry allowing the addition of two different ligands [200], (**C**) base-labile and photolabile protecting groups allow the successive combination of two different ligands [201].

**Figure 8:**
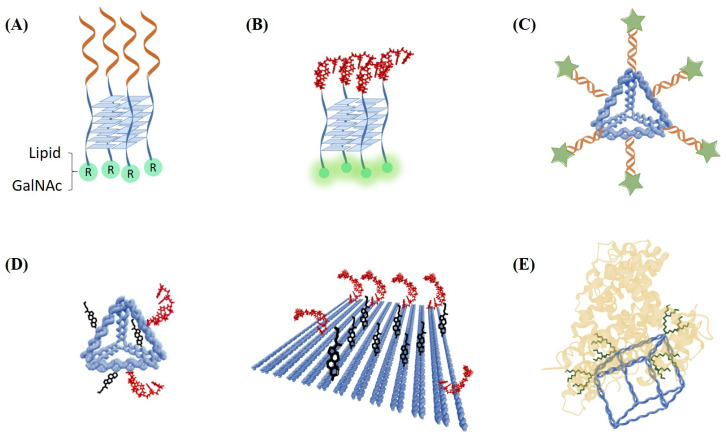
Schemes of DNA nanostructures carrying multifunctional ligands to improve drug delivery. (**A**) G-quadruplex functionalized with ASOs and hydrophobic groups [202,203], (**B**) G-quadruplex functionalized with FdU_n_ oligomers [204], (**C**) DNA tetrahedron with four siRNAs carrying folic acid [205], (**D**) DNA tetrahedron and DNA origami functionalized with cholesterol moieties to deliver floxuridine [206], and (**E**) DNA cube with oligonucleotides carrying dendritic alkyl chains being able to modulate the affinity to human serum albumin [207].

Successful developments in the field of lipid- and peptide-oligonucleotide conjugates, as well as the achievement of the therapeutic use of the triantennary GalNAc siRNA modification, have sparked the progress of multifunctional oligonucleotide conjugates. The ligands can be identical, as seen in the triantennary GalNAc, different, such as diverse peptides or lipids, or both in the same oligonucleotide (Figure 7A). The multifunctionalization can be achieved by the incorporation of several ligands in one oligonucleotide (Figure 7) or by hybridization of several monofunctionalized oligonucleotides in simple or complex DNA nanoassemblies (Figure 8).

One of the first studies showed that the conjugation of three histidine-rich peptides enhanced the efficacy of antisense oligonucleotides [208]. Peptides which include bombesin peptide sequences for receptor targeting are covalently linked to a splice switching antisense oligonucleotide. The multifunctional conjugates were more effective than conjugates containing only one bombesin peptide. A second study demonstrated that the addition of two peptides in internal positions of an antisense oligonucleotide targeting BRAF V600E oncogene increased target recognition and stability to exonuclease degradation [209]. A tridentate derivative carrying three cyclic RGD peptides have been developed and incorporated into siRNAs showing an increase in inhibitory properties [124]. In recent research, novel reagents that allow the incorporation of multiple peptides by click chemistry have been developed [200]. In addition, they allow the preparation of heterofunctional conjugates through a clever use of click chemistry and amino-protection (Figure 7B) [200]. Similarly, thiol and amino-linkers carrying base-labile and photolabile protective groups were designed in order to add different ligands in the same oligonucleotide (Figure 7C) [201]. Recently, aminooxy click chemistry has been used for the preparation of building blocks to synthesize oligonucleotides carrying two equal or different ligands [210].

Interestingly, the assembly of monofunctionalized oligonucleotides generates multifunctional structures. For example, three oligonucleotides designed to form a triplex were functionalized with a short coiled peptide that interacts between them, thereby stabilizing the triplex structure [211]. Lipids conjugated to guanine rich oligonucleotides are also of great interest. AS1411, a nucleolin-binding aptamer capable to fold into multiple mono- and bimolecular G-quadruplex, has been seen to form nanoaggregates when conjugated to lipids facilitating the delivery of anticancer or antiviral agents [212,213]. Similarly, G-quadruplex formation is shown to address the assembly of two peptide strands generating two-loop structures on top of the G-quadruplex. This approach can be used with homo and hetero peptide sequences [214]. G-rich oligonucleotides designed to form parallel G-quadruplex functionalized with hydrophobic groups (Figure 8A) are able to tetramerize, which results in a multifunctionalized G-quadruplex with affinity to viral proteins [74,75,76] and/or cell membranes [202,203].

Moreover, advances in nanotechnology offer solutions to the challenge of therapeutic oligonucleotides delivery. DNA nanostructures allow the simple generation of molecularly-defined multifunctionalized therapeutic drugs, as they are biocompatible and can be programmed in different sizes (in the range of 20 nm (DNA tetrahedron and DNA cube) to 120 nm of DNA origami) and shapes [215,216,217]. DNA nanostructures used as drug delivery vehicles incorporate nucleic acids drugs and target ligands within the structure itself. Among them, DNA tetrahedra [218] have been studied to show excellent results in terms of drug-loading and drug release [219]. For example, DNA tetrahedron has been functionalized with four siRNAs carrying folic acid [205], which resulted in increased therapeutic properties (Figure 8C). Similarly, a DNA tetrahedron carrying four units of a cationic amphipathic peptide was prepared to deliver doxorubicin to mitochondria [220]. DNA tetrahedron and DNA origami (Figure 8D) were also assembled to prepare defined nanodrugs to deliver floxuridine functionalized with up to eight molecules of cholesterol, which demonstrates the beneficial properties of cholesterol in terms of enhanced cellular uptake [206]. In another study, the assembly of a DNA nanocube (Figure 8E) and oligonucleotides carrying dendritic alkyl chains allowed the preparation of nanocubes carrying defined hydrophobic sites being able to modulate the affinity of the DNA cube to human serum albumin [207]. The resulting hydrophobic nanocubes have increased serum stability. In a more complex way, the addition of several units of the iron transporter protein transferrin into a planar DNA origami [221] resulted in protein-DNA origami complexes with higher cytoplasmatic uptake, compared to unmodified structures.

### 2.6. Oligonucleotide Conjugates Currently in Advanced Preclinical or Clinical Trials

Several oligonucleotide conjugates are being analyzed in the initial phases of clinical studies. Table 2 summarizes some examples that have been mentioned in a recent bibliography. Although the information is fragmented, the activity in this field is intensive. Most of the pharmaceutical companies working in therapeutic oligonucleotides include a large investment in the development of targeting molecules to improve their clinical outcome, and are thrilled by the success of the GalNAc modification. The incorporation of peptides into phosphorodiamidate morpholino oligomers (PMO) for the treatment of hereditary neuromuscular diseases such as DMD or Myotonic Dystrophy type 1 (DM1) is one of the most studied subjects. These conjugates, known as PPMOs (peptide-PMO, [165]), are exon-skipping antisense oligonucleotides that modulate RNA splicing aiming to skip the mutated exon that causes the disease. Some unconjugated PMO oligomers have been approved for DMD human treatment but, in some cases, low activity and poor delivery to muscle have been described. For these reasons, PPMOs, such as SRP-5051 (Sarepta), PGN-EDO51 (PepGen) or ENTR-601-44 (Entrada), are being extensively studied for DMD treatment [197]. DM1 is also a target in these studies, although there is not an oligonucleotide approved for human use [222]. PGN-EDODM1 (Entrada) targets the inhibition of the dystrophia myotonia protein kinase (DMPK) gene, while ENTR-701-CUG (Entrada) targets the muscleblind like splicing regulator (MBNL) protein, by binding to the CUG repeat. Both carry a peptide component, an EDO (enhanced delivery oligonucleotide) or an EEV (endosomal escape vehicle) peptide [197]. Pip6a-PMI-CAG7 (Oxford University) is a PPMO that combines a PMO and the cellular penetrating peptide Pip6a (Table 1), which promotes an occupancy-based mechanism for MBNL protein and prevents the binding of the toxic CUG repeat [222].

Imetelstat (GRN163L, Geron) is a lipid-oligonucleotide conjugate with palmitic acid at the 5′-end that is designed to inhibit telomerase activity [223]. Currently, a phase III clinical trial has finalized with positive results for the treatment of myelofibrosis [224].

ARG520-HBV (Arrowhead) is a 1:1 mixture of two cholesterol-siRNAs against Hepatitis B virus (HBV). The cholesterol moiety is used to enhance delivery to hepatocytes. Phase II clinical trials demonstrate good pharmacokinetic properties in a single-dose study [224]. Recent studies show that ARC-520 is active in HBV patients; but absolute Hepatitis B antigen reduction is moderate [225].

## 3. Conclusions

Drugs based on nucleic acids are capturing a large interest in the pharmaceutical field due to the recent successes on the development of unique and safe drugs for several hereditary and metabolic diseases. However, some challenges remain, the most important being the development of specific formulations to deliver the oligonucleotide active compound to the target cells and tissues. Both peptide and lipid-DNA conjugates have been studied to solve the delivery issue. Since the early 1990s, during the development of the antisense technology, different strategies to prepare them have appeared.

At the beginning, lipids were thought to act as passive hydrophobic cellular entry facilitators, but the role of cellular receptors was soon discovered. Recently, it has been described that extrahepatic delivery by oligonucleotide-lipid conjugates is possible, especially for oligonucleotides aimed to act in muscle and the central nervous system.

On the other hand, peptide-oligonucleotide conjugates are difficult to synthesize because of the incompatibility of the protection schemes; nevertheless, efficient postsynthetic conjugate chemistries, as well as stepwise approaches, are effective in the production of relatively large amounts that are needed for clinical studies. Several oligonucleotide-peptide conjugates are being translated to clinical evaluation with increased activity. PMO-peptide conjugates for Exon-skipping therapies and PNA-peptide conjugates as potential antibiotics are also intensively considered.

Hetero- or homo-bi/trifunctional conjugates carrying lipid and/or peptides have been prepared showing interesting properties such as increased affinity and higher potency. Furthermore, DNA nanostructures are promising compounds for the preparation of defined multifunctional drugs offering the possibility of preparing molecularly homogeneous nanostructures carrying several drugs and/or delivery and targeting agents.

## Figures and Tables

**Table 1:** Selected sequences of peptides, especially cell-penetrating peptides, that are studied for enhanced oligonucleotides delivery. The two main cellular mechanism of delivery used are direct penetration or energy-dependent endocytosis, described in the main text. Once internalized, some peptide sequences facilitate the endosomal escape.

Name	Sequence	Type	Application	Ref.
**Hydrophobic**				
P4	LGAQSNF	ASO (PS)	SSO(2OMe) for DMD	[140]
**Polycationic**				
TAT	RKKRRQRRR	siRNA	Neurodegenerative pathologies Lung and Malignant glioma	[141,142,143]
		ASO (PNA/PMO)	bacteria & nematodecell lines	[144,145]
pAnt	RQIKIWFQNRRMKWKKGGC	ASO (PNA/PS)	bacteria & nematodecell lines	[129,144]
PolyArg	Rn (*n* = 6–18) optimal 8 to 10	siRNA	Neurons and different cancers	[146,147]
(KFF)_3_K	KFFKFFKFFK	ASO (PNA/LNA)	Anti-bacterial	[144,148]
(RXR)_4_	RXRRXRRXRRXRXR	ASO (PMO)	Anti-viral anti-bacterial	[144,149,150,151,152]
B peptide	(RXRRBR)_2_XB	ASO (PMO)	SSO of DMD DM1	[153,154,155,156,157,158]
Pip5e	RXRRBRRXR-ILFQY-RXRBRXRB	ASO (PMO)	SSO of DMD, SMA	[159]
Pip6a	RXRRBRRXR-YQFLI-RXRBRXRB	ASO (PMO)	SSO of DMD, SMA	[160,161,162,163]
Pip6b	RXRRBRRXR-IQFLI-RXRBRXRB	ASO (PMO)	SSO of DMD, SMA	[164,165]
**Amphipathic**				
MPG-8	GALFLGFLGAAGSTMGAWSQPKKKRK	siRNA	Xenograft tumor model	[166]
		ASO	Mammalian cells	[167]
Pep-1	KETWWETWWTEWSQPKKRK	siRNA	Cells	[168]
CADY	GLWRALWRLLRSLWRLLWRA	siRNA	Several cancer cells	[169]
		ASO	Anti-bacterial	[170]
RICK	KWLLRWLSRLLRWLARWLG	siRNA	H glioblastoma cells	[171]
599	GLFEAIEGFIENGWEGMIDGWY(G)_4_(R)_9_K	siRNA	Oral cancer	[172,173]
Pepfect6	AGYLLGK(ε-Mtt)INLKALAALAKKIL	siRNA	Cell lines (various)	[174]

ASO, antisense oligonucleotide; SSO, splice-switching oligonucleotides; DMD, Myotonic dystrophy; DM1, Myotonic dystrophy type J; SMA, spinal muscular atrophy; PS, phosphorothioate; PNA, peptide nucleic acids; PMO, phosphorodiamidate morpholinos; LNA, locked nucleic acid, 2OMe, 2′-O-methyl; X, 6-aminohexanoic acid; B, beta-alanine.

**Table 2:** Oligonucleotide conjugates in recent advanced preclinical and clinical studies.

Therapeutic Target/Disease	Name	Conjugate	Sponsor	Status
DMD, Exon51	SRP-5051	Peptide-PMO	Sarepta therapeutics	Phase II
DMD, Exon 51	PGN-EDO51	Peptide EDO-PMO	PepGen	Phase I
DMD, Exon 53	PGN-EDO53	Peptide EDO-PMO	PepGen	preclinical
DM1, DMPK	PGN-EDODM1	Peptide EDO-PMO	PepGen	preclinical
DMD, Exon 44	ENTR-601-44	Peptide-PMO	Entrada therapeutics	precilinal
DMD, Exon 44	ENTR-601-45	Peptide-PMO	Entrada therapeutics	precilinal
DM1	ENTR-701-CUG	Peptide EEV-PMO	Entrada therapeutics	precilinal
DM1, MBNL1	Pip6a-PMI-CAG7	CPP peptide-PMO	Oxford University	preclinical
Telomerase	GRN163-L, imetelstat	Palmitate-ASO	Geron Corporation	Phase III
Chronic Hepatitis B	ARC-520-HBV	Two cholesterol-siRNAs	Arrowhead pharmaceuticals	Phase II

CPP, cell-penetrating peptide; DM1, Myotonic Dystrophy type 1; DMD, Duchenne Muscular Dystrophy; DMPK, dystrophia myotonia protein kinase; EDO, enhanced delivery oligonucleotide; EEV, endosomal escape vehicle; MBNL1, muscleblind like splicing regulator 1.

## Data Availability

Not applicable.
